# The Inclusion of LGBTQ+ Health across the Lifespan in Pre-Registration Nursing Programmes: Qualitative Findings from a Mixed-Methods Study

**DOI:** 10.3390/healthcare11020198

**Published:** 2023-01-09

**Authors:** Michael Brown, Edward McCann, Brian Webster-Henderson, Fidelindo Lim, Freda McCormick

**Affiliations:** 1 School of Nursing and Midwifery, Queen’s University Belfast, 97 Lisburn Road, Belfast BT9 7BL, UK; 2 School of Nursing and Midwifery, Trinity College Dublin, D02 PN40 Dublin, Ireland; 3 School of Health and Psychological Sciences, Nursing Department, City, University of London, Myddelton Square, London EC1R 1UW, UK; 4 Deputy Vice Chancellor, University of Cumbria, Fusehill Street, Carlisle CA1 2HH, UK; 5 Rory Meyers College of Nursing, New York University, 433 1st Avenue, New York, NY 10010, USA

**Keywords:** education, inclusion, LGBTQ, lifespan, nursing, older persons, students, transgender

## Abstract

Poor physical and mental ill-health is experienced by many LGBTQ+ people, compounded by a reluctance to access healthcare services. This reluctance is attributed to experiences of heteronormative assumptions and negative attitudes encountered. Despite increasing recognition of the need to include LGBTQ+ health in undergraduate healthcare programmes, inconsistencies and gaps in content, skills development, and assessment are still apparent. The aim of the study was to identify LGBTQ+ health content within nursing and midwifery pre-registration programmes and identify education best practice and innovation. A mixed-methods study involving a quantitative and qualitative design was undertaken. The qualitative findings from a nursing perspective were extracted from the dataset for further detailed analysis and are reported in this paper. Information about the study and an online survey were distributed to 135 Schools of Nursing and Midwifery in the UK and Ireland. Individual semi-structured online interviews took place with academics. Qualitative data from 29 survey responses and 12 follow-up interviews were thematically analysed. Eight of the 12 follow-up interviews were held with nursing academics and following data analysis three themes emerged: (i) LGBTQ+ health across the lifespan; (ii) taking the initiative for LGBTQ+ health inclusion; and (iii) identifying and overcoming challenges. The findings highlight the endeavours by nursing academics to integrate LGBTQ+ health within pre-registration programmes to meet the education needs of students and the opportunity to develop curriculum content to address the needs and concerns of LGBTQ+ people across the lifespan.

## 1. Introduction

To ensure inclusivity of the range of sexual and gender identities, including gay, lesbian, bisexual, transgender and people who identify as queer, questioning, asexual and intersex, the term LGBTQ+ will be used in this paper. There is a global focus on providing healthcare that is equitable and accessible to all citizens, including those who identify as LGBTQ+ [1,2]. While policy aspirations are welcome and necessary, many LGBTQ+ people continue to experience significant health needs and health inequalities with barriers to accessing healthcare appropriate to their needs, requiring sustained attention to improve health and well-being [3,4]. These issues raise concerns regarding wider social justice, social inclusion, human rights and discrimination, compounded by lack of knowledge and skills on behalf of some health professionals regarding the distinct health needs of LGBTQ+ people [5,6].

## 2. Background

In the LGBT+ Pride 2021 Global Survey of 27 countries, responses showed that 80% identify as heterosexual, 3% as gay, lesbian or homosexual, 4% as bisexual, 1% as pansexual or omnisexual, 1% as asexual, 1% as “other”, and 11% do not know or did not disclose [7]. Despite the prevalence of LGBTQ+ population globally and the focus on their addressing human rights and ensuring equality in some countries, many continue to be invisible and, in some countries, live in fear of their lives [8]. Consequently, in some countries, LGBTQ+ people experience fear and significant barriers accessing healthcare appropriate to their needs for fear of stigma, victimisation, discrimination and further marginalisation [9]. These experiences can have a significant impact on the health well-being of LGBTQ+ people and may lead to minority stress. The minority stress model highlights the consequences and the potentially negative impact of internalised homophobia, transphobia and biphobia [10,11]. The negative and hostile societal reactions to LGBTQ+ people can result in psychological distress and ill-health including depression, drug-use, anxiety and suicidality [12]. Minority stress can therefore have important and long-term implications and consequences on the physical and mental health and lives of LGBTQ+ people [13].

There is a well-developed research evidence-base of the specific health needs and concerns experienced by LGBTQ+ people. Mental health conditions are also prevalent such as anxiety, depression, self-harm, suicidal ideation, attempted suicide, eating disorders and drug and alcohol use [14,15]. From a physical health perspective, some LGBTQ+ subpopulations are at increased risk of conditions such as HIV, sexually transmitted infections, hepatitis [16]. LGBTQ+ people are not a homogeneous group and their health profile and consequently the health needs of the subpopulations, such as gay men, lesbians, bisexuals and transgender people differ, necessitating specific care and supports [17,18]. Additionally, the health profile and health needs differ across the lifespan, with the needs of LGBTQ+ youth being different, for example, from those of seniors [19]. Therefore, service and education responses required need to be age and subpopulation specific to ensure culturally competent care and support [20].

Consequently, due to the scope and extent of their health concerns, LGBTQ+ people require access to a range of health services, including primary care, mental health and sexual health [21]. Yet, despite their needs, many report bias, negative and discriminatory experiences from professionals when accessing health services [22,23]. Research studies evidence heteronormative assumptions in healthcare and education delivery, impacting on the attitudes, knowledge and skills of students to effectively meet the needs of LGBTQ+ people as an integral part of their practice [24,25]. A recent review of the international research evidence highlighted the connection between religious beliefs and the views held by student health professionals, notably among some Christians and Muslims. The situation is compounded by the religious attitudes of some nurse educators that effects the design and delivery of the curriculum [26]. Therefore, ensuring the consistent delivery of culturally competent LGBTQ+ health curriculum that is sensitive care and support that fully reflects the health concerns and needs of LGBTQ+ people is aspirational [25]. In response and ensure that the health workforce, including nurses, are prepared with the attitudes, knowledge and skills necessary to provide culturally competent, person-centred healthcare a sustained education and practice development focus is required [27]. However, despite the evidence of the scope and extent of the health inequalities and health needs and discrimination within health services experienced by many LGBTQ+ people, education materials, resources and education interventions specific to their needs, they are not utilised and implemented [28,29]. There are therefore important roles for higher education and health services to ensure that students and the wider workforce receive education and practice development that reflects the care and support needs of LGBTQ+ people [30]. There is growing research evidence of the positive impact and benefits of delivering culturally sensitive and competent education for all student health professionals and practitioners including nurses, midwives, psychiatrists and physicians [31,32]. Therefore, the aim of this study was to identify the views and experiences of nurse academics in the provision of LGBTQ+ health content within pre-registration nursing programmes in the United Kingdom and the Republic of Ireland.

## 3. Materials and Methods

### 3.1. Design

A mixed-methods design was utilised for this study with the first stage involving an anonymous online survey and the second stage semi-structured individual interviews. The qualitative findings from a nursing perspective were extracted from the dataset for further detailed analysis and are reported in this paper. Prior to completing the survey, respondents were provided with information about the study, with the option at the end of the survey to opt-in to take part in a follow-up qualitative interview. Selected respondents were sent a participation information leaflet and consent form which were completed prior to the interview. The survey identified LGBTQ+ education activity and best practice taking place in Schools of Nursing and Midwifery across the UK and Ireland. The interviews with the nursing academics provided a deeper understanding and insight into education innovations in the delivery of LGBTQ+ health to nursing students.

### 3.2. Participants

All Heads of Schools of Nursing and Midwifery in the UK and Ireland (*n* = 135) were contacted by email and provided with information about the study. To avoid perceived coercion, the information was forwarded by an administrator to academics for completion. From October 2020 to March 2021, a total of *n* = 29 (39%) responses were received from Schools of Nursing and Midwifery across the participating countries. Consent to be contacted to participate in a follow-up individual semi-structured interview was provided by 21 respondents from which a sample of 12 academics across nursing and midwifery agreed to participate. Of them, eight represented nursing programmes from across England, Scotland, Wales, Northern Ireland and the Republic of Ireland, with their views and experiences providing the data for this paper.

### 3.3. Data Collection

The online anonymous survey using Microsoft Forms was ‘live’ between 30 October 2020 and 31 March 2021. The survey comprised 36 items, including demographic details and a focus on LGBTQ+ health concerns within nursing and midwifery pre-registration programmes. Some questions had a choice of responses—“non-existent”, “limited”, “adequate”, “moderately adequate” and “fully adequate”. Whilst other questions asked for preferences to be rated in order of importance with a free text option for further details. For stage two the research team developed and piloted a semi-structured interview guide to obtain clarity and depth of LGBTQ+ health within programmes. The interview questions were open-ended to prompt participants to provide detailed responses regarding current curriculum activity and best education practice. Interviews took place between 25 February and 13 April 2021 and lasted between 30 and 55 min. Interviews were recorded and transcribed verbatim then anonymised by assigning each participant a gender-neutral pseudonym and removing all identifiable information.

### 3.4. Data Analysis

The data were imported to Microsoft Excel for analysis, with the free text qualitative elements extracted as part of the analysis process [33]. The research team read each transcript separately to gain an understanding of the participants’ views and experiences. The analysis of the data was facilitated by the data management programme NVivo 12. As part of the analysis process, themes and subthemes were systematically identified [34]. The approaches used in the qualitative data analysis and synthesis were rigorously followed to ensure the trustworthiness, dependability and credibility throughout the process [35,36]. Consequently, the qualitative views and experiences of nurse academics regarding LGBTQ+ health education in nursing pre-registration programmes were extracted from the larger dataset for further detailed analysis to allow for the findings to inform future nurse education delivery [37].

## 4. Results

Three themes emerged following analysis of the qualitative data that were cognisant with nursing care and support: (i) LGBTQ+ health across the lifespan; (ii) taking the initiative for LGBTQ+ health inclusion; and (iii) identifying and overcoming challenges.

### 4.1. LGBTQ+ Health across the Lifespan

The continued visibility of the diverse LGBTQ+ population and the ability to integrate their health needs and concerns within nursing programmes was important to the nursing academics. Attempts to incorporate the health concerns of LGBTQ+ people across the lifespan from early years to later life was a challenge encountered by most participants. However, whilst equality and diversity focus on, for example, disabled people and other minority groups, participants suggested that the needs of LGBTQ+ people required greater consideration and development to ensure it is embedded more fully within nursing programmes.

The academics were mindful that LGBTQ+ people across the lifespan often encountered a range of unique individual experiences that changed at different points across the lifespan when accessing healthcare. There was an appreciation and sensitivity particularly for older LGBTQ+ people as they may have lived in an era when their sexual orientation was illegal resulting in secrecy and hesitancy when seeking healthcare. Conversely, in a ‘joined up learning environment’ there was also recognition that a lot can be learned from students and their recent experiences of social change regarding LGBTQ+ people.

*“The current health and social care service is not inclusive of many of these people, it’s not. It is like getting people to acknowledge that and say well you are the leaders of the future, so how are you going to start thinking about it in your practice so when you are in practice how are you going to be more aware of it. I think there’s a lot of lip service paid towards people who identify as lesbian, gay, bisexual, transgender, intersex, non-binary.”*

(Alex)

*“There is a population of children and young people, and indeed adults, who identify as being on the autistic spectrum and also within itself because of autism and identity, that led to gender identity challenges that they had as well.”*

(Finn)

*“There is a whole range of older people out there with a different lifestyle or different types of lifestyles or their different backgrounds and you should be considering them in what you do as much as anyone else.”*

(Sam)

### 4.2. Taking the Initiative for LGBTQ+ Health Inclusion

In the absence of a defined curriculum, the participants had often taken the initiative to develop and integrate LGBTQ+ health content and embed it within their nursing programmes. There was a consensus that there should be opportunities for openness and discussion to equip students with knowledge and skills to support and understand the health needs and concerns of LGBTQ+ people across the lifespan. There was a notable move towards smaller groups rather than standard lectures and it was apparent that this approach may encourage some of the students who identity as LGBTQ+ to reflect and share their experiences, thereby supporting learning.

The academics identified and sourced a range of resources to support the delivery within their programme. These included collaborating with academic colleagues, health professionals from local services, and members of the LGBTQ+ community. A private chat facility on an online platform for students to engage with academics to request further LGBTQ+ information and awareness had been introduced in one institution. The assessment of LGBTQ+ health knowledge and skills were identified by some of the participating nurse academics as absent from both formative and summative student assessments such as group work, OSCEs, assignments and examinations; this was viewed as an area for future development. As a result, some participants had included an LGBTQ+ option for students to choose in clinical scenarios, assessments, or assignments.

*“I think it is just really important that students see themselves reflected within the curriculum. And students see the patients that they are going to meet reflected within the curriculum. If your curriculum is really traditional, it is whitewashed, everybody is heterosexual from a middle-class background, that kind of thing, it is not reflective of real life and it’s not reflective of your student population either. You have to have an inclusive curriculum so that students can see themselves reflected within it.”*

(Chris)

*“... making sure that everything isn’t always from a cis-gender heteronormative slant. That’s one way of just integrating it so not having a specific ‘this is your LGBT day, where you learn about this’. It is across the curriculum, you are giving scenarios, you are giving patient examples, or that kind of thing of using people who are not hetero-sexual and bringing that into the mix.”*

(Taylor)

*“We always make sure in our assessments where there is any kind of clinical scenario or consideration of patient management pathway that the LGBTQ+ community is also included as an option for students to perhaps answer a question on that.”*

(Finn)

### 4.3. Identifying and Overcoming Challenges

It was appreciated by the participants that LGBTQ+ health may be seen as a ‘taboo’ and sensitive subject for some nursing students, with some academics feeling uncomfortable or poorly prepared to deliver. This included introducing the challenges experienced by some LGBTQ+ people within health services and effective strategies to raise and approach sensitive issues and confidently answer questions. Most of the participating academics had embraced the subject due to personal interest and experiences, whilst others who delivered content within their programme did so reluctantly, describing a lack of confidence as the primary reason.

While some nurse academics endeavoured to include life stories from the LGBTQ+ communities, there was recognition of the needs of ageing LGBTQ+ people, viewed by some participants as a ‘tricky thing’. This was often situated within the experiences of older LGBTQ+ people who had experienced historical stigma and discrimination due to their sexuality, leading to a lack of trust in health services per se. Others in contrast had been ‘out’ for many years and due to ageing and impaired cognitive ability required to access care and support and felt it necessary to hide their sexual identity for fear of further discrimination. Participants recognised the importance of providing students an insight and understanding of the range of issues and challenges experienced by some LGBTQ+ people and their role in supporting equality of access to healthcare and confronting discrimination. Transgender people and those in the process of transitioning were identified as a population with new and emerging concerns with a need for students to be ‘sensitive when they come across it in everyday practice’. Therefore, most nurse academics sought to be proactive and integrated information in their programmes which included scenarios and opportunities for students to engage in discussion, conversations and self-reflection in a safe learning environment.

*“When you are working with older people, you don’t know what baggage they are bringing with them. You don’t know what their history is … many have suffered because of stigma, prejudice and discrimination. Therefore, if that’s what they have suffered, why should they trust you now … Someone that’s transitioned earlier in life, and now if they have got dementia, it may mean that they don’t recognise their genitals or they wonder why they are being called by a name that wasn’t the name assigned at birth, that type of thing.”*

(Joe)

*“We have also included trans scenarios in there because that is the one scenario that people feel very uncomfortable about as they don’t know how to deal with it. We also provided staff training to allow us to embed transgender issues throughout the curriculum. Most specifically we incorporated that into our scenarios for consideration of what it means to be trans and not everyone has had or will even have surgery.”*

(Finn)

*“Sometimes I start off with talking about different countries and the legal timeline for when things become legal … it is important when you are thinking about doing your teaching to put it in the context of all these actual larger structural barriers that have been in place as well.”*

(Taylor)

## 5. Discussion

The findings from the current study evidence the efforts by nursing academics to include LGBTQ+ health concerns within pre-registration nursing programmes. There is evidence of positive initiatives developed by academics who try and embed the topic within the curriculum, despite some feeling a lack confidence. Examples were provided of LGBTQ+ theory integration, health needs evidence and the assessment of learning and skills development. Despite the positive developments, a range of further developments were identified by nursing academics that would enhance and embed the inclusion of LGBTQ+ health within programmes.

### 5.1. Implications for Policy and Regulation

Whilst this study did not explicitly seek to include experiences of LGBTQ+ curriculum issues from a regulator or policy perspective, there are nevertheless factors that require further careful consideration by regulators, arm’s length bodies, and professional organisations in relation to our findings.

#### 5.1.1. Regulators

A range of LGBTQ+ knowledge and skills by students in learning or registered nurses on further development qualifications are required as part of their educational experience. However, a distinct lack of clear LGBTQ+ focused competencies, or outcomes are currently evident in their absence. In the UK, the Nursing and Midwifery Council as the regulator of nurses, midwives and health visitors clearly outlines its equality, diversity, and inclusivity approach within its organisational strategy and highlights a range of professional expected behaviours and requirements within its code of practice [38,39]. However, there is an omission of *clear* and *explicit* statements of expectation in relation to LGBTQ+ knowledge or skills for all learners within regulatory standards and expected outcomes. A clear mandate not masked within the language of equality, diversity and inclusivity or expected behaviours is required to make a significant change in the learning experience of nursing and midwifery students. This may have relevance for other regulators of nursing globally.

#### 5.1.2. Policy Makers

Meyer suggests that legislative policy is and should be required to facilitate better support and education for all, across the educational sphere in relation to a range of issues associated with those who identify as LGBTQ+ [40]. However, any policy developments need to be research informed and led which can be a challenge in relation to funding for those undertaking research and for others to see the importance of findings and action required.

*“We need courage, allies, and strong leaders to help our research inform policy.”*

[40]

The alignment of policy, practice and pedagogy are critical to make significant enhancements not only to the learning and practice experiences of students, many of whom may personally identify as part of the LGBTQ+ community. Those responsible for the organisations, in which students learn, have a leadership role to play in policy development and implementation, thereby ensuring that discriminatory practices, bullying, harassment, intolerance, homophobia, abuse are all clearly aligned to make a clear stand and articulate appropriate actions as part of their role in creating a culture for both academics and students that signal inclusivity in action [41].

#### 5.1.3. Policy Influencers

Advancing a progressive and inclusive approach to LGBTQ+ issues in policy also needs the engagement of policy influencers. Within the profession of nursing a range of professional, charity, policy, research or practice focused organisations exist that can have an influence on policy makers. The policy influencers, or those who can challenge the world in which policy makers often base their thinking and ideas, can be instrumental in creating cultures of change [42]. Stonewall, a leading UK LGBT charity founded in 1989 with an original focus of challenging and changing policy, at that time Section 28 in the UK which forbid teachers to educate pupils regarding LGBT issues had global success in introducing change. There are therefore important opportunities for policy makers and influencers regarding the wider LGBTQ+ social justice and inclusion agenda as suggested in a study conducted in the United States of America [43]. Pedagogical curriculum writers in nurse education have therefore the potential to shape and influence change in regulation, curriculum content, delivery, assessment and awareness.

Our evidence demonstrates the positive activities regarding the LGBTQ+ visibility and learning experience within nursing curricula across the UK and Ireland, with opportunities to learn from the positive international experiences [44,45]. However, wider research points to the limited UK evidence regarding effective strategies to address and respond to LGBTQ+ health concerns [4]. Consequently, there is a need to ensure that developments that support further change and for influencers to share education and practice innovations internationally to ensure that those who regulate, oversee or approve curricula can do so from an insightful LGBTQ+ lens [46]. Failure to do so will increase and not reduce the health inequalities experienced by many LGBTQ+ people.

### 5.2. Implications for Practice

Findings of the current study provide further impetus for sustained collaboration between academia and clinical practice owing to bi-directional influences and synergies of these domains [24]. Academics, professional development leaders, and students are envisioned to be co-creators of LGBTQ+ curriculum that synthesise authentic patient narratives and evidence-based practices.

The four conceptual perspectives, minority stress model, life course, intersectionality, and social ecology, set forth by the National Academy of Medicine in the United States (formerly Institute of Medicine), can guide the implementation of LGBTQ+ inclusive practice [47]. Practitioners are therefore encouraged to explore the burden of minority stress on the patient’s healthcare scenario, understand the impact of the patient’s experiences from an earlier development stage on the next stage, examine the confluence of an individual’s multiple identities and how they interact, and address the layers of social influences that affects health and well-being. With the growing research focus on the experiences and needs of transgender people and educating and preparing nurses regarding their distinct needs, it is particularly relevant and contemporary given the recognition of the impact of minority stress [48].

The well-known health disparities experienced by LGBTQ+ populations are closely linked with discrimination and not intrinsic personal characteristics related to sexual orientation, gender identity, or intersex status [49]. Practitioners must take into considerations that the health disparities experienced by LGBTQ+ individuals are unevenly distributed. For example, due to historic and lingering social and economic marginalisation, suicidality, HIV infection, smoking, and drug and alcohol use are disproportionately higher among transgender populations [50]. For these reasons, transgender health requires special attention. Across all levels and fields of practice, teams need to apply the *Standards of Care for the Health of Transgender and Gender Non-Conforming People* created by the World Professional Association for Transgender Health [51]. Practitioners can for example access the *Guidelines for the Primary and Gender-Affirming Care of Transgender and Gender Nonbinary People*, published by the University of California, San Francisco [52].

The care of LGBTQ+ populations remains largely focused on pathology, with an overdue opportunity to consider a well-being perspective in examining LGBTQ+ health across the lifespan. Nurses, academics, and nursing students who have taken on the initiative of becoming champions of LGBTQ+ health need to be supported by wider stakeholders. For example, the formation of a LGBTQ+ employee resource group can serve as a forum for mentorship, professional development, and advocacy [53]. The improvement of practice outcomes for LGBTQ+ populations will be contingent on the enduring collaboration between clinical practice partners, academia and community-based organisations.

### 5.3. Implications for Education

Ensuring that LGBTQ+ visibility and content within the nursing curriculum is a key component of breaking down barriers, challenging and addressing heteronormative assumptions, and working toward an inclusive curriculum based on a noticeable societal representation of communities within the learning experience of nursing students [54]. Our evidence highlights the imperative that LGBTQ+ knowledge, experiences and representation is required within a nursing curriculum that provides real world representation and relevance [24]. However, there are several challenges to this, both real and perceived. There may be a perception that regulated curriculum are crowded and challenging to address all learning needs and issues a nurse in learning may encounter. This often results in curricula that is based on fundamental themes or concepts that can achieve the regulated and required outcomes which in turn shape the curriculum design which can be later applied to a range of real-life scenarios [55]. It has been suggested that nursing curriculum content is oversaturated by addition of new content or new concepts [56]. However, the many aspects of LGBTQ+ health concerns need to be respectfully included within a nursing curriculum design, that does not lead to over saturation. Rather, what needs to be addressed is an under-representation of LGBTQ+ health concerns within the nursing curriculum that do not reflect the societies in which nurses practice and deliver nursing care and support and in which student nurses learn [57].

The current study seeks to build on and address these concerns to ensure that the nursing curricula whether it leads to nurse registration or contributes to a continuing professional development context, represent in the learning approaches, the outcomes, the pedagogic activities in which nurses operate and practice within and are reflective of recent international studies and evidence reviews [20,24,30,31,56]. Based on our findings and the wider evidence, there is a need for appropriate approaches to learning, representing and developing new knowledge and skills within a curriculum and should have an integrated approach to LGBTQ+ society, needs, challenges and requirements [54]. This is further espoused by Gobbi’s theory of “the hidden curriculum” which suggests that while an issue may not be fully understood, and how to conceptualise it, or integrate within curricula, does not indicate inaction. The theory describes nursing practice as a bricoleur activity, one that has many shades and aspects, like a collage that requires us to seek new knowledge, new approaches and new skills to “get the job done” [58]. Challenges such as lack of confidence, lack of knowledge, limited personal experience of LGBTQ+ health concerns are cited as issues that contemporary academic pedagogy needs to address in the *bricoleur* of an inclusive curriculum. Gobbi argues that the acquisition of new knowledge, new insights and new experiences can address these challenges [58]. Furthermore LGBTQ+ health as part of an inclusive curriculum should be no respecter of field of nursing or indeed focus of the aspect of nursing practice, such as mental health, children’s nursing, intellectual disability nursing, adult nursing, as LGBTQ+ knowledge, skills, values and relevance are important to all [59].

From a curriculum design perspective, this can and should be threaded throughout the learning journey for all students. Bidell highlights the importance of incorporating LGBTQ+ health into the curriculum from the outset woven into a range of pedagogical approaches and content; clinical skills scenarios, interprofessional learning, appropriate aspects of social science, life science and pharmacology as well as small group learning, reflective learning situations with students based on their practice learning journeys [60]. Our findings suggest there is merit in educationalists sharing good practice, supporting innovative ideas in pedagogical design and learning from what has and has not worked well as a network to help embed LGBTQ+ concepts, relevance and inclusivity within the nursing curricula, a recommendation supported by international research evidence [61,62,63]. This can be readily achieved in an online format which facilitates the sharing of good practice to support and shape evolving curricula.

### 5.4. Implications for Research

Researchers of LGBTQ+ health in nursing are unanimous in their assertion for the need to step up cross-cutting efforts to address the unique healthcare issues of the LGBTQ+ population in research [23,64,65]. A 2018 scoping review reported that 33 articles (0.19%) on LGBTQ+ health were published between 2009 and 2017 in 20 nursing journals with the highest impact factors [66]. This finding reflects an increase, albeit small, in nursing publications on LGBTQ+ health compared with a similar study in 2010 [64].

Given that some of the academics in the present study took it upon themselves to integrate LGBTQ+ health, in the absence of a defined curriculum, a potential research topic is to explore in depth the barriers and facilitators (for example, personal, work climate, systems factors) in championing this work.

While there is significant data on the lack of and inconsistent integration of LGBTQ+ health in nursing education across all levels [61], more research is needed to appraise effective strategies in teaching this topic. Studies comparing the traditional lecture format versus small group seminars, preferred by some participants of the current study, is warranted. An area of interest for educational research is examining the use of active learning modalities and instructional technologies to best deliver LGBTQ+ health content.

Although the lifespan perspective is recognised as a model for addressing the healthcare needs of gender and sexual minorities, the predominance of studies done on gay adults and mostly white subjects is apparent [47]. Participants of the current study have recognised the need for situated learning experiences in caring for vulnerable LGBTQ+ older adults and transgender population. Researchers are encouraged to conduct studies involving LGBTQ+ subpopulations with identities that intersect with race, ethnicity, socioeconomic status, geographic location, disability, age, religion, immigration status, and other factors [47].

An important area of research is developing a validated instrument for self-assessment by members of nursing academia (foe example, leadership, faculty, clinical partners, and practitioners) regarding LGBTQ+ inclusive practices. The purpose of this tool would be to assess knowledge, attitudes, and behaviours that foster LGBTQ+ inclusivity based on individual role in academia. An LGBTQ+ inclusivity self-assessment tool developed by the Centers for Disease Control and Prevention in the United Stated for use by practitioners working with LGBTQ+ youth may serve as a prototype in developing one for nurse educators [67].

### 5.5. Strengths and Limitations

The current study adds significantly to the evidence-based research concerning the distinct needs and requirements of LGBTQ+ people. The views and experiences of nurse academics was sought related to the provision of LGBTQ+ health content contained within pre-registration nursing programmes in the UK and Ireland. The results of the study provide enlightening insights into education innovations utilised in the delivery of LGBTQ+ health content to nursing students. Although the response rate may be considered low, important lessons have been learned in relation to the subject and opportunities identified for future research endeavours.

## 6. Conclusions

The study has identified important issues related to LGBTQ+ health content and nurse education programmes in the UK and Ireland, including substantive teaching and learning considerations. The interviews provided opportunities for participants to share their views and experiences of LGBTQ+ specific issues within current nursing curricula including innovations and challenges. There are very clear implications in terms of nursing policy and practice and the players involved in the potential co-creation of appropriate curriculum content and future educational developments. LGBTQ+ health should be effectively threaded through the diverse range of teaching and learning approaches. Research opportunities exist to evaluate and appraise such educational initiatives. Through the provision of culturally competent and responsive education for nursing students, and allied health professionals, this in turn will have positive benefits and enhanced therapeutic outcomes for people who identify as LGBTQ+ and significant people in their lives.

## Data Availability

Not applicable.
